# Morphometric variability among the species of the Sordida subcomplex (Hemiptera: Reduviidae: Triatominae): evidence for differentiation across the distribution range of *Triatoma sordida*

**DOI:** 10.1186/s13071-017-2350-y

**Published:** 2017-09-06

**Authors:** Julieta Nattero, Romina Valeria Piccinali, Catarina Macedo Lopes, María Laura Hernández, Luciana Abrahan, Patricia Alejandra Lobbia, Claudia Susana Rodríguez, Ana Laura Carbajal de la Fuente

**Affiliations:** 1 Facultad de Ciencias Exactas y Naturales, Departamento de Ecología, Genética y Evolución/Laboratorio de Eco-Epidemiología, Universidad de Buenos Aires, Ciudad Autónoma de Buenos Aires, Buenos Aires, Argentina; 2 CONICET - Universidad de Buenos Aires, Instituto de Ecología, Genética y Evolución de Buenos Aires (IEGEBA), Ciudad Autónoma de Buenos Aires, Buenos Aires, Argentina; 3 Laboratório Interdisciplinar de Vigilância Entomológica em Diptera e Hemiptera- Instituto Oswaldo Cruz, Avenida Brasil, 4365, Manguinhos, Rio de Janeiro, 21040-900 Brazil; 4 Centro Regional de Investigaciones Científicas y de Transferencia Tecnológica de La Rioja (CRILAR-CONICET), Entre Ríos y Mendoza s/n, 5301 La Rioja, Argentina; 5 Centro de Referencia de Vectores, Ministerio de Salud de la Nación, Hospital Colonia, Pabellón Rawson calle s/n, X5164 Santa María de Punilla, Córdoba Argentina; 6 Intituto de Investigaciones Biológicas y Tecnológicas, CONICET, FCEFyN, UNC, Avenida Vélez Sarsfield 299, X5000JJC Córdoba, Argentina

**Keywords:** Sordida subcomplex, *Triatoma garciabesi*, *Triatoma guasayana*, *Triatoma patagonica*, *Triatoma sordida*, Head, Pronotum, Wing, Linear morphometrics, Geometric morphometrics

## Abstract

**Background:**

The Sordida subcomplex (Triatominae) comprises four species, *Triatoma garciabesi*, *T. guasayana*, *T. patagonica* and *T. sordida*, which differ in epidemiological importance and adaptations to human environments. Some morphological similarities among species make taxonomic identification, population differentiation and species delimitation controversial. *Triatoma garciabesi* and *T. sordida* are the most similar species, having been considered alternatively two and a single species until *T. garciabesi* was re-validated, mostly based on the morphology of male genitalia. More recently, *T. sordida* from Argentina has been proposed as a new cryptic species distinguishable from *T. sordida* from Brazil, Bolivia and Paraguay by cytogenetics. We studied linear and geometric morphometry of the head, wings and pronotum in populations of these species aiming to find phenotypic markers for their discrimination, especially between *T. sordida* and *T. garciabesi*, and if any set of variables that validates *T. sordida* from Argentina as a new species.

**Results:**

Head width and pronotum length were the linear variables that best differentiated species. Geometric morphometry revealed significant Mahalanobis distances in wing shape between all pairwise comparisons. *Triatoma patagonica* exhibited the best discrimination and *T. garciabesi* overlapped the distribution of the other species in the morphometric space of the first two DFA axes. Head shape showed differentiation between all pairs of species except for *T. garciabesi* and *T. sordida.* Pronotum shape did not differentiate *T. garciabesi* from *T. guasayana*. The comparison between *T. garciabesi* and *T. sordida* from Argentina and *T. sordida* from Brazil and Bolivia revealed low differentiation based on head and pronotum linear measurements. Pronotum and wing shape were different between *T. garciabesi* and *T. sordida* from Brazil and Bolivia and *T. sordida* from Argentina. Head shape did not differentiate *T. garciabesi* from *T. sordida* from Argentina.

**Conclusions:**

Wing shape best delimited the four species phenotypically. The proposed cryptic species, *T. sordida* from Argentina, differed from *T. sordida* from Brazil and Bolivia in all measured shape traits, suggesting that the putative new species may not be cryptic. Additional studies integrating cytogenetic, phenotypic and molecular markers, as well as cross-breeding experiments are needed to confirm if these three entities represent true biological species.

**Electronic supplementary material:**

The online version of this article (10.1186/s13071-017-2350-y) contains supplementary material, which is available to authorized users.

## Background

**Fig. 1:**
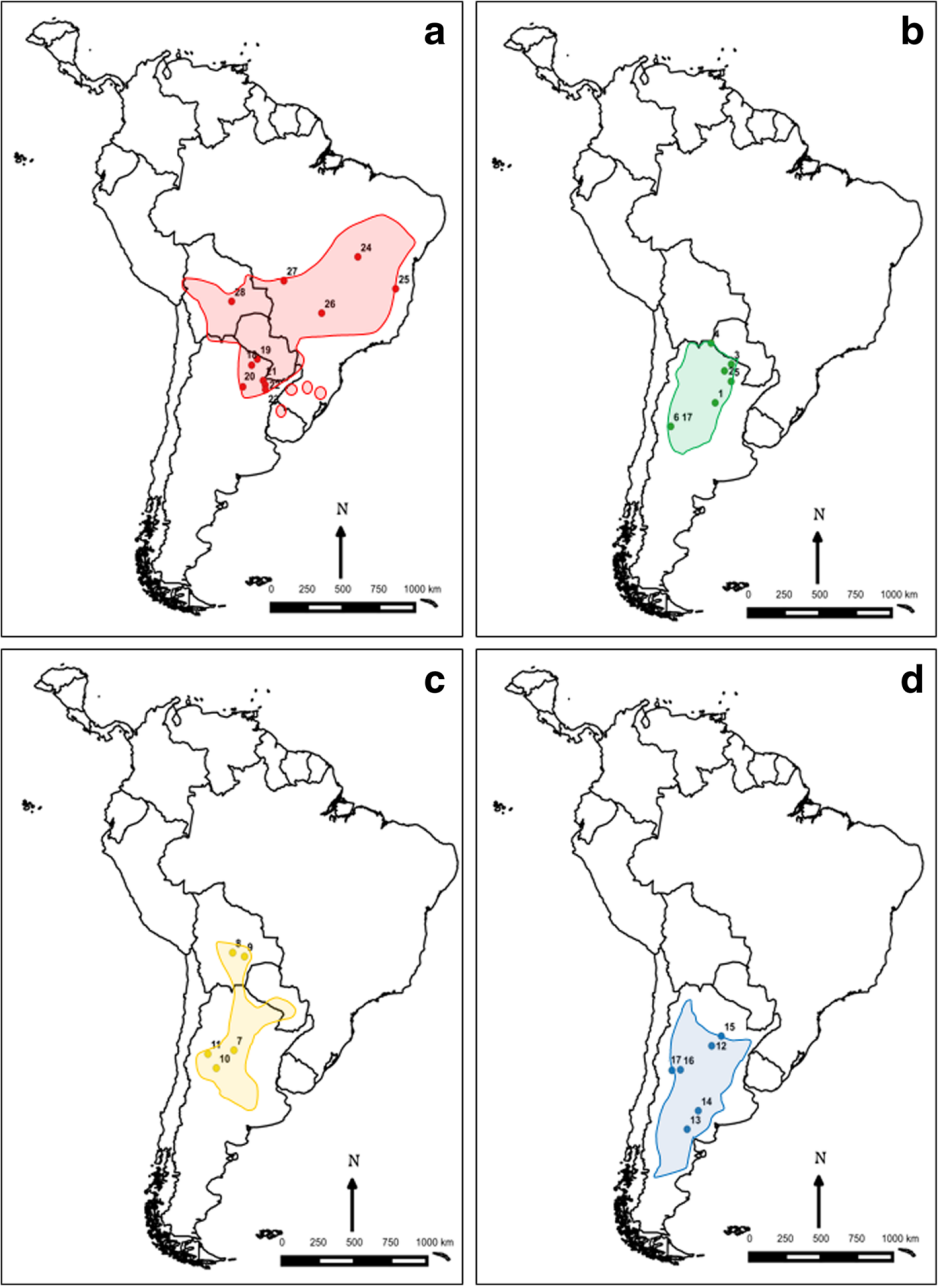
Geographical maps and locations of the studied populations of *Triatoma sordida* (**a**), *T. garciabesi* (**b**), *T. guasayana* (**c**) and *T. patagonica* (**d**). Population codes are as in Table 1. Distribution areas were based on [56]

**Fig. 2:**
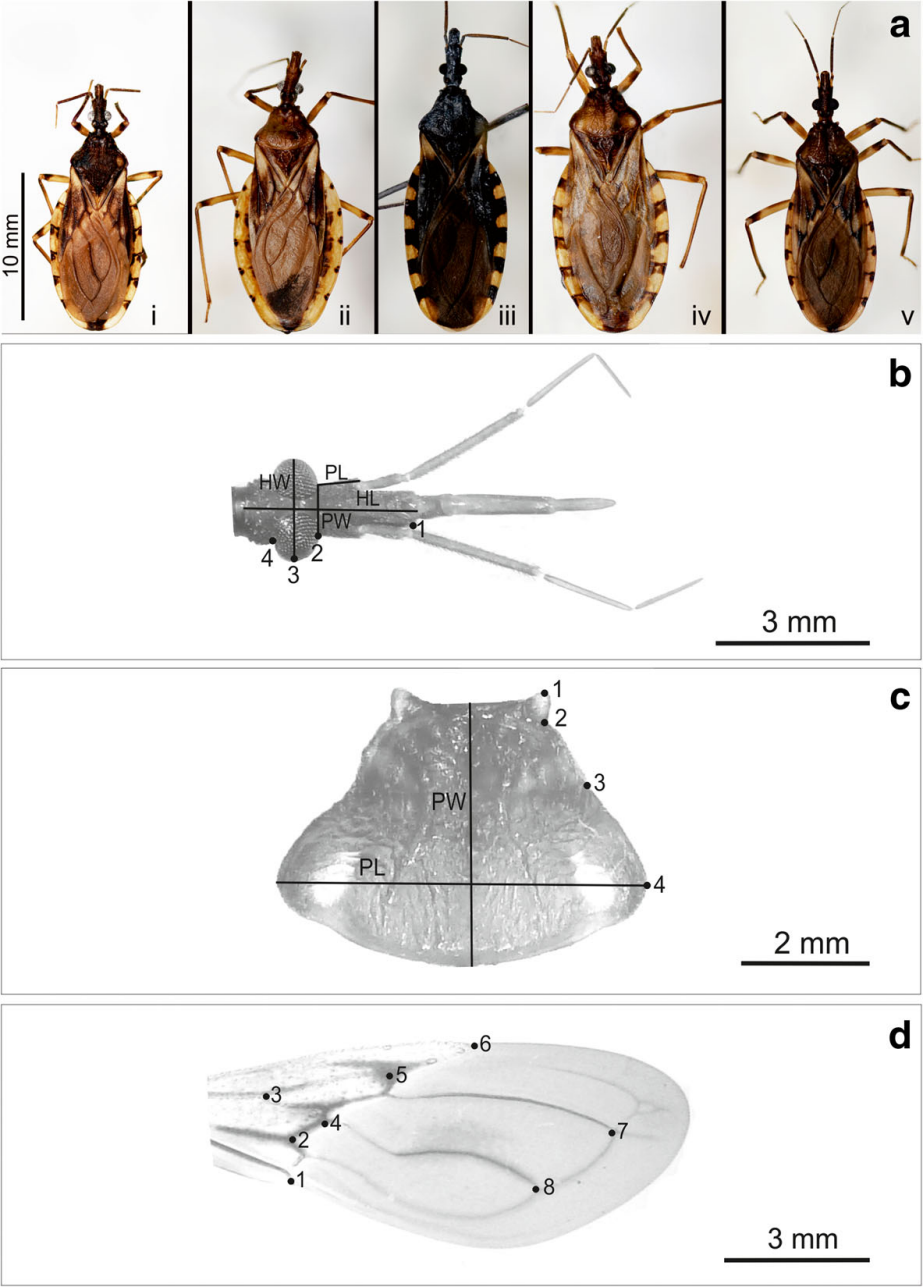
**a** Specimens of *Triatoma garciabesi* (**i**), *T. guasayana* (**ii**), *T. patagonica* (**iii**) and *T. sordida* (**iv**) from Argentina and *T. sordida* from Brazil (**v**). Linear measurements and landmark positions used for the head (**b**), pronotum (**c**) and forewings (**d**) of the four species included in this study. **b** Head width (HW), preocular length (PL), head length (HL), preocular width (PW), landmark positions 1–4. **c** Pronotum width (PW), pronotum length (PL), landmark positions 1–4. **d** Forewings, landmark positions 1–8

The subfamily Triatominae (Reduviidae) consists of 151 described species grouped in 15 genera, widely and mainly distributed in the American continent, including the Caribbean Islands (reviewed in [1]). All the species of this subfamily are obligate haematophagous that can transmit *Trypanosoma cruzi* Chagas, 1909, the etiological agent of Chagas disease [1]. *Triatoma* is the most conspicuous genus within the subfamily, with 84 species grouped in 8 complexes and 14 subcomplexes [1]. The Sordida subcomplex traditionally included four species: *Triatoma sordida* Stål, 1859; *T. patagonica* Del Ponte, 1929; *T. guasayana* Wygodzinsky & Abalos, 1949; and *T. garciabesi* Carcavallo, Cichero, Martínez, Prosen & Ronderos, 1967, with differences in their distribution areas (Figs. 1 and 2a), epidemiological importance and level of adaptation to the human environment [2]. *Triatoma sordida* is the species of highest epidemiological importance, considering *T. cruzi* infection rates and its ability to colonize human-made or modified habitats, mainly chicken coops [3–6]. This species has a wide distribution in Argentina, Uruguay, Paraguay, Bolivia and Brazil (Figs. 1a and 2a). *Triatoma garciabesi* occupies northwestern and central Argentina (Figs. 1b and 2a), with recent records in southern Bolivia and western Paraguay [7]. It is rare in human habitats and is commonly associated with bird nests in wild environments. However, it has been collected from peridomestic structures like chicken coops [8, 9]. *Triatoma guasayana* is found in Argentina, Bolivia and Paraguay (Figs. 1c and 2a); it is sylvatic and invades houses temporally [8, 10]. *Triatoma patagonica* is endemic to Argentina; it is mainly sylvatic but has also been found colonizing domiciles and peridomiciliary structures in several Argentine provinces (Figs. 1d and 2a) [11–13]. A detailed taxonomic comparison of *T. guasayana*, *T. patagonica* and *T. sordida* using morphometric measurements, genitalia and antennal structures was presented elsewhere [14].

*Triatoma sordida* and *T. garciabesi* exhibit high morphological similarity; they partially overlap in their geographical distribution and were considered a single species until 1967, when *T. garciabesi* was described based on specimens from central Argentina [15]. However, *T. garciabesi* and *T. sordida* were considered synonymous during two decades [16]. In 1998, *T. garciabesi* was re-validated as a species based on characteristics of microhabitats, male genitalia and cytogenetics [17]. To identify these two species, linear measurements from head and pronotum structures have been traditionally used [18]. In addition, cytogenetic, isoenzyme and molecular studies have shown that Argentine *T. sordida* populations (*T. sordida* Arg) differ from those of other areas of its distribution (*T. sordida* Brazil and Bolivia) [7, 19, 20]. This evidence has led to the recent proposal of considering *T. sordida* Arg a new species [7].

Linear and geometric morphometry has been widely used as a useful tool to delimit species, subspecies and intraspecific variation within the subfamily Triatominae [21–26]. The most commonly used phenotypic markers to resolve variations in geometric morphometry are those for the wings [27], although markers for the head have also been used effectively in morphometric studies [21, 23]. For example, wing geometric morphometrics was successfully applied to delimit species within the *T. brasiliensis* [28] and *T. dimidiata* [29] species complexes, with results being in agreement with those of molecular systematics studies [30, 31]. To our knowledge, the pronotum, has been used to evaluate the dispersive capacity of other species of the Triatominae [32, 33], but not to differentiate closely related species, subspecies or varieties.

Given the morphological similarities of the four species of the Sordida subcomplex, the partial overlapping of their distribution, the differences in their epidemiological importance and the proposal of *T. sordida* Arg to be considered a new cryptic species, in this work we studied the morphometry of the head, wing and pronotum modules using linear and geometric morphometry variables. We included populations of the four species that cover a broad part of their distribution area in Argentina, Brazil and Bolivia, aiming to find phenotypic markers that distinguish the four species, especially the closest ones, *T. sordida* and *T. garciabesi*. Finally, we intended to determine if these modules have some morphological variables that validate the proposal to consider *T. sordida* Arg a new species.

## Methods

### Insects

A total of 255 males from 28 populations of *T. garciabesi*, *T. guasayana*, *T. patagonica* and *T. sordida* from Argentina, Brazil and Bolivia were included in this study (Table 1, Figs. 1a-d and 2a). Only males were evaluated because their sample size was larger than that of females, and because previous studies suggest sexual dimorphism at least for wing shape in *T. sordida* [4]. In addition, male individuals from Patiño, Balbuena, Aguirre, El Colchon and most *T. patagonica* populations are the same as those used for recent cytogenetic studies [7]. Only populations with at least four males were included. Most of the populations (23 of 28) were collected from the field, whereas the remaining were colonies with no more than three generations reared in the laboratory (Table 1). Insects from colonies were supplied by the “Centro de Referencia de Vectores (CeReVe)” from the National Health Ministry of Argentina. All populations were collected from peridomestic structures, except those from “Reserva Natural Bosques Telteca”, Mendoza Province, which were collected from sylvatic habitats.

**Table 1:** Geographical location and coordinates, origin and number of individuals of the studied populations of *Triatoma garciabesi*, *T. guasayana*, *T. patagonica* and *T. sordida*

Species	Population code	Locality	Province/State	Country	Origin	Latitude	Longitude	No. of individuals
*T. garciabesi*	1	Aguirre	Sgo del Estero	Argentina	Field	-29.52	-62.17	10
	2	Balbuena	Chaco	Argentina	1st LG	-25.64	-60.93	10
	3	Patiño	Formosa	Argentina	1st LG	-24.83	-60.03	5
	4	Santa Victoria E	Salta	Argentina	Field	-22.27	-62.71	14
	5	El Triangulo	Chaco	Argentina	Field	-26.93	-60.05	12
	6	Reserva Telteca	Mendoza	Argentina	Field	-32.38	-68.06	6
*T. guasayana*	7	Sobremonte	Córdoba	Argentina	Field	-29.76	-64.05	5
	8	Mataral	Cochabamba	Bolivia	Field	-18.11	-64.21	14
	9	Tita	Santa Cruz	Bolivia	Field	-18.58	-62.69	9
	10	San Martín	La Rioja	Argentina	Field	-31.82	-66.38	9
	11	Independencia	La Rioja	Argentina	Field	-30.27	-67.46	8
*T. patagonica*	12	Mitre	Sgo del Estero	Argentina	3th LG	-29.41	-62.79	9
	13	Avellaneda	Río Negro	Argentina	3th LG	-39.53	-66.05	10
	14	Utracán	La Pampa	Argentina	3th LG	-37.28	-64.57	8
	15	El Nochero	Santa Fe	Argentina	Field	-29.68	-61.51	9
	16	Santa Rosa	San Luis	Argentina	Field	-32.32	-66.93	12
	17	Reserva Telteca	Mendoza	Argentina	Field	-32.38	-68.06	7
*T. sordida*	18	El Colchón	Chaco	Argentina	Field	-25.61	-60.36	5
	19	Crucero Belgrano	Formosa	Argentina	Field	-24.87	-59.61	22
	20	El Nochero	Santa Fe	Argentina	Field	-29.68	-61.51	4
	21	Corrientes	Corrientes	Argentina	Field	-27.47	-58.83	5
	22	San Miguel	Corrientes	Argentina	Field	-28.00	-58.57	5
	23	San Roque	Corrientes	Argentina	Field	-28.57	-58.54	5
	24	Combinado	Tocantins	Brazil	Field	-12.51	-46.33	13
	25	Itaobim	Mina Gerais	Brazil	Field	-16.34	-41.33	13
	26	Paranaiba	Mato Grosso	Brazil	Field	-19.29	-51.11	12
	27	Várzea Grande	Mato Grosso	Brazil	Field	-15.38	-56.10	9
	28	Santa Cruz	Santa Cruz	Bolivia	Field	-17.87	-63.00	5

*Abbreviation*: *LG* laboratory generation

In some populations of *Triatoma guasayana,* brachypterous individuals were reported to occur (J. Espinoza, personal communication). We considered this event and evaluated measures of the pronotum and wings to ensure that brachypterous individuals were not included in this study.

Species were identified following a dichotomous key traditionally used for this purpose [18]. Populations from Patiño and Balbuena were originally identified as *T. sordida*; however, based on cytogenetic and molecular characteristics, these two populations were identified as *T. garciabesi* [7] and were considered as such in this work.

Digital images of ventral and dorsal views of the head, and dorsal views of the pronotum and wings of each individual were taken using a digital camera (Lumix DMC-ZS7, Panasonic) connected to a stereomicroscope (Stemi SV-11, Carl Zeiss). Images include the reference scale.

### Linear morphometry

Images of head and pronotum of each individual were processed using the UTHSCSA ImageTool for Windows ver. 3.00 [34]. Total area (mm^2^) and four linear measurements for the head and two for the pronotum (mm) were taken (Fig. 2b, c). Head length was measured from the base of the head to the anteclipeum and head width corresponded to the maximum external width of head in dorsal view. Preocular length was measured from the base of the anteniferous tubercules to the eyes and preocular width was measured at the anterior base of eyes. Pronotum length and pronotum width were the maximum length and width, respectively. Head width, pronotum length, and head and pronotum area did not show a normal distribution and were normalized using Log_10_ transformation for statistical analysis.

### Geometric morphometry

A landmark-based approach was applied to study geometric morphometrics of heads, pronotums and right forewings. Four coplanar type II landmarks of ventral view of the head, four type II landmarks of the pronotum and eight type I landmarks of the wings were defined and collected using TPS dig 2.17 [35] for each individual (Fig. 2b-d). Ventral view of the head was used to minimize error digitalization because it is more planar than dorsal view. For the head and pronotum, the average of the landmarks on both sides (four landmarks) was used to reduce intraindividual variation and minimize digitization errors [36].

Shape variables were obtained through the generalized Procrustes analysis superimposition algorithm and the subsequent projection of the Procrustes residuals into a Euclidean space [37]. Both non-uniform and uniform components [38] were used as shape variables. These two components describe the differences in shape as deviations from an average configuration of landmarks. The uniform component describes global variation such as stretching and compression, and the non-uniform component corresponds to local variation [39]. The isometric estimator centroid size (CS) derived from the landmark based analysis was used as a measure of overall size [40].

### Statistical analysis

We analyzed linear and geometric variables of the three modules, i.e. head, pronotum and wings, separately. Species were grouped in two different ways for the analyses. One clustering considered interspecific comparisons, i.e. *T. garciabesi*, *T. guasayana*, *T. patagonica* and *T. sordida* from Argentina, Brazil and Bolivia as a single species. The other group included comparisons among closely related taxa, i.e. *T. garciabesi*, *T. sordida* Arg and *T. sordida* Brazil and Bolivia as three different taxa (Table 1). Brazilian and Bolivian *T. sordida* populations were grouped because a phylogenetic analysis showed that they belong to a unique clade separated from the Argentine population (see figure 3 in [7]).

**Fig. 3:**
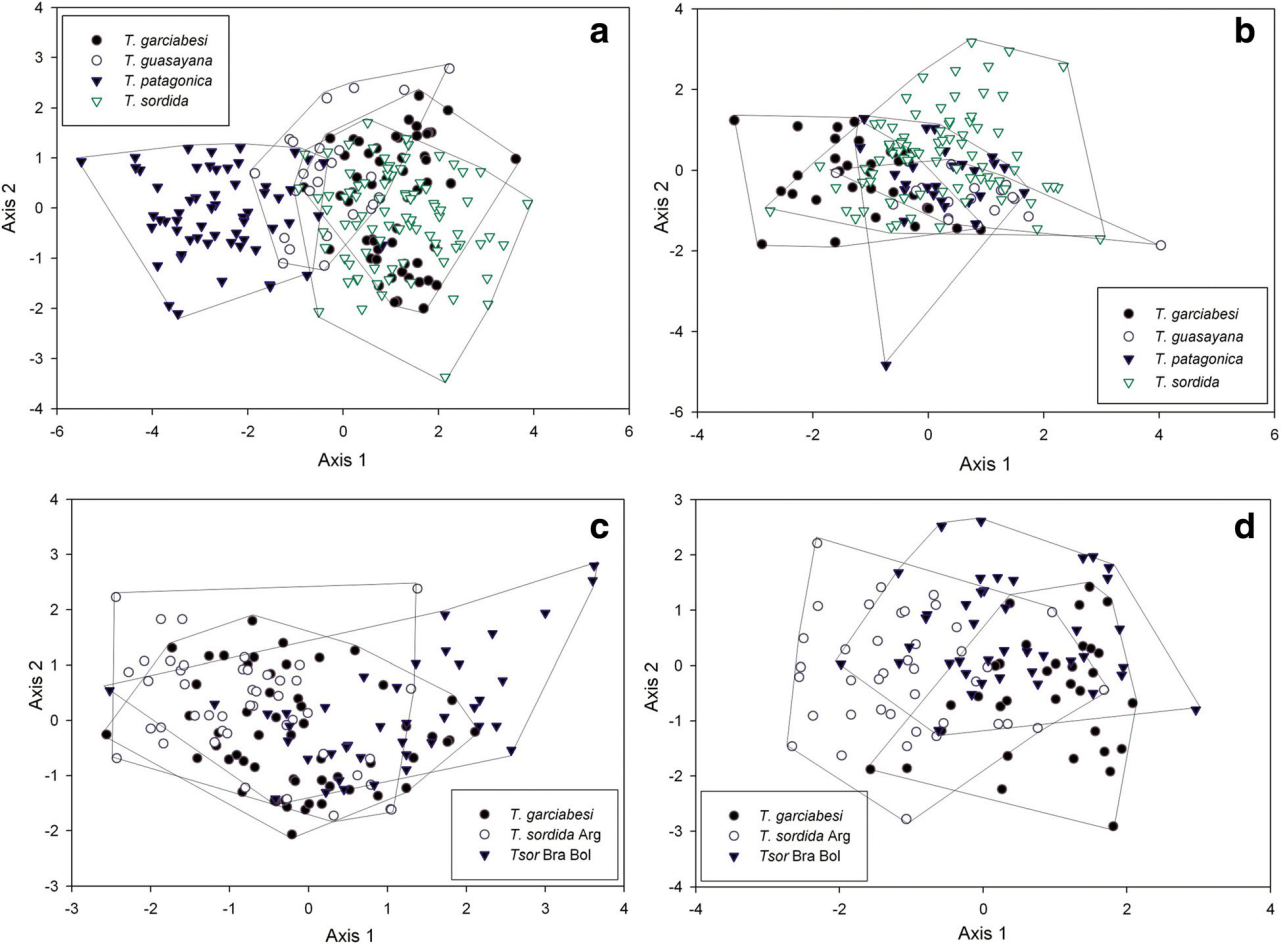
Factorial maps in the plane of the two first axes of discriminant function analysis for linear measurements of the head and pronotum in populations of *Triatoma garciabesi*, *T. guasayana*, *T. sordida* and *T. patagonica*. For easy visualization, the lines connect the most external individuals of each population. Centroids are represented with the same symbol that identifies each species. **a** Head measurements of the four species. **b** Pronotum measurements of the four species. **c** Head measurements of *T. garciabesi*, *T. sordida* Argentina and *T. sordida* Brazil and Bolivia. **d** Pronotum measurements of *T. garciabesi*, *T. sordida* Argentina and *T. sordida* Brazil and Bolivia

We performed Discriminant Function Analysis (DFA) per module for linear and shape measurements for both groups (i.e. interspecific comparisons and comparisons among closely related taxa, see above). For shape measurements, Mahalanobis distances between pairs of species were calculated and their significance was evaluated using a non-parametric test based on permutations (1000 runs). We represented the Mahalanobis distances between pairs of species or closely related taxa in neighbor joining (NJ) trees. The percentage of phenotypic similarity between pairs of species was calculated using the cross-check test of discriminant analysis [40, 41].

The relationship between CS and shape discrimination among groups (allometry) was estimated using a multivariate regression between the Procrustes coordinates (dependent variables) and the CS (independent variable). This analysis was performed for each module and for both groups. These regressions showed a significant association (*P* < 0.01) in all groups of comparisons except for wings from *T. garciabesi*, *T. sordida* Arg and *T. sordida* Brazil and Bolivia. Since the allometric effect was small for wings (1.8%) and heads (2.8%), the DFA analyses were performed with the original Procrustes coordinate values. For the pronotum, the regression confirmed a strong allometric effect, accounting for 18.30% (81.70% for the non-allometric component) of the total shape variance for the comparison of the four species and 19.23% (80.77% for the non-allometric component) for the comparison of *T. garciabesi*, *T. sordida* Arg and *T. sordida* Brazil and Bolivia. For these cases, the residual values from the multivariate regression analysis were used to investigate shape variation independent of size, i.e. the non-allometric component [42].

For comparing overall wing size among species, the CS was used in an ANOVA with a *post-hoc* Tukey’s tests. The software InfoStat [43] was used for linear analysis and the software MorphoJ [44], the CLIC 98 package (http://mome-clic.com) and JMP v. 6.0.0 (SAS Institute Inc., 2005) for shape variables.

## Results

### Linear morphometric analysis

#### Interspecific comparisons

DFA performed for head linear measurements of the four species showed that the first two discriminant factors explained 96.89 and 2.70% of the total variation, respectively. Of the five linear measurements included in the DFA, head width was the one that best discriminated between species for the first two axes. The factorial distribution map of each individual in the space of the first DFA axes showed that *T. patagonica* was the only highly differentiated species (86% of the correctly assigned individuals) (Fig. 3a, Table 2). The other three species overlapped in their distribution in the morphometric space and had low percentages of assignment (38–56%), with 41% of *T. garciabesi* individuals being assigned to *T. sordida* and 33% of *T. sordida* individuals being assigned to *T. garciabesi*. *Triatoma guasayana* had 12 to 16% of individuals assigned to the other three species (Table 2).

**Table 2:** Reclassification of *Triatoma garciabesi* (*T. gar*), *T. guasayana* (*T. gua*), *T. patagonica* (*T. pat*) and *T. sordida* (*T. sor*) performed for linear variables of the head and pronotum. The number and percentage of assigned individuals derived from discriminant function analyses are presented

Species	No. of individuals	*T. gar*	*T. sor*	*T. gua*	*T. pat*	Module
		*n* (%)	*n* (%)	*n* (%)	*n* (%)	
*T. gar*	56	21 (38)	23 (41)	12 (21)	0 (0)	Head
	33	21 (64)	6 (18)	2 (6)	4 (12)	Pronotum
*T. sor*	88	29 (33)	39 (44)	19 (22)	1 (1)	Head
	83	15 (18)	40 (48)	18 (22)	10 (12)	Pronotum
*T. gua*	25	3 (12)	4 (16)	14 (56)	4 (16)	Head
	19	1 (5)	1 (5)	13 (68)	4 (21)	Pronotum
*T. pat*	57	0 (0)	1 (2)	7 (12)	49 (86)	Head
	24	4 (17)	7 (29)	6 (25)	7 (29)	Pronotum

The first discriminant factor of the DFA performed for pronotum measurements explained 70.29% of total variation, with pronotum length being the variable that best discriminated species along this axis. The second axis, explained mostly by pronotum area, explained 28.79% of the total variation. The factorial map showed low differentiation between species (Fig. 3b), with *T. guasayana* showing the lowest misclassification error followed by *T. garciabesi* (32 and 36%, respectively, Table 2). About half of the *T. sordida* individuals (52%) were incorrectly assigned, but only 18% were attributed to *T. garciabesi*. Unlike DFA results for head, the results for pronotum yielded the highest percentage of misassigned *T. patagonica* individuals (71%).

#### Comparisons among closely related taxa

The first two axes of the DFA for head measurements explained the whole variation (94.62 and 5.38% for first and second axes, respectively). Length of preocular region discriminated best these entities. The factorial map showed low discrimination between groups in this space (Fig. 3c), with *T. garciabesi* exhibiting 45% of the individuals assigned to *T. sordida* Arg, as well as the highest misclassification error (62.50%, Table 3). *Triatoma sordida* Brazil and Bolivia showed 39% of misclassified individuals, with 37% being assigned to *T. garciabesi*. For *T. sordida* Arg. 26% of the individuals were assigned to *T. garciabesi* and only 6% to *T. sordida* Brazil and Bolivia. For pronotum measurements, the first two DFA axes explained the whole variation (75.30 and 24.70% for first and second axes, respectively) with pronotum area being the variable that better discriminated groups. The factorial map did not show a good differentiation between groups (Fig. 3d). However, misclassification error improved for *T. sordida* Arg (26%) and *T. garciabesi* (36%) in comparison with head DFA results (Table 3). For *T. sordida* Brazil and Bolivia misclassification error was 51%.

**Table 3:** Reclassification of the closely related taxa *T. sordida* from Argentina (*T. sor* Arg), *T. sordida* from Brazil and Bolivia (*T. sor* BB) and *T. garciabesi* (*T. gar*) performed for linear variables of the head and pronotum. The number and percentage of assigned individuals derived from discriminant function analyses are presented

Species	No. of individuals	*T. gar*	*T. sor* Arg	*T. sor* BB	Module
		*n* (%)	*n* (%)	*n* (%)	
*T. gar*	56	21 (38)	25 (45)	10 (18)	Head
	33	21 (64)	4 (12)	8 (24)	Pronotum
*T. sor* Arg	47	12 (26)	32 (68)	3 (6)	Head
	42	5 (12)	31 (74)	6 (14)	Pronotum
*T. sor* BB	41	15 (37)	1 (2)	25 (61)	Head
	41	10 (24)	11 (27)	20 (49)	Pronotum

### Geometric morphometric analysis

#### Interspecific comparison

**Fig. 4:**
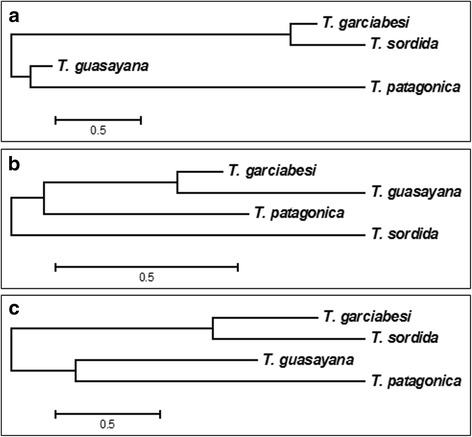
Neighbor-joining trees derived from Mahalanobis distances of shape measurements for the head, pronotum and wings in populations of *T. garciabesi*, *T. guasayana*, *T. sordida* and *T. patagonica*. **a** Head measurements. **b** Pronotum measurements. **c** Wing measurements

The first two axes of the DFA for head shape variation explained 96.92 and 1.96%, respectively. Mahalanobis distances across the four species showed significant differences between all pairs of species except for *T. sordida* and *T. garciabesi* (Table 4). When Mahalanobis distances were used to build a NJ tree, these two species appeared as the most similar, followed by *T. guasayana* (which was equally separated from all the other species) and *T. patagonica* which was the most distant species from *T. sordida* and *T. garciabesi* (Fig. 4a). In addition, *T. sordida* and *T. garciabesi* showed the lowest percentage of correctly assigned individuals derived from the DFA analysis (63% for *T. sordida* and 53% for *T. garciabesi*, Table 5) and appeared completely overlapped in the factorial map (Additional file 1: Figure S1). No individual of these two species was assigned to *T. patagonica* nor was any individual of *T. patagonica* assigned to *T. sordida* or *T. garciabesi* (Table 5).

**Table 4:** Mahalanobis distances between pairs of species performed for shape variables of the head, pronotum and wings. Interspecific comparison: *Triatoma garciabesi* (*T. gar*), *T. guasayana* (*T. gua*), *T. patagonica* (*T. pat*) and *T. sordida* (*T. sor*). Comparison among closely related taxa: *T. sordida* from Argentina (*T. sor* Arg), *T. sordida* from Brazil and Bolivia (*T. sor* BB) and *T. garciabesi* (*T. gar*)

Group	Pairs of species	Head	Pronotum	Wing
*T. gar, T. gua, T. pat, T. sor*	*T. gar* * vs* * T. gua*	2.01***	0.64	2.71***
	*T. gar* * vs* * T. pat*	3.84***	1.11**	3.07***
	*T. gar* * vs* * T. sor*	0.58	1.49***	1.22***
	*T. gua* * vs* * T. pat*	2.07***	1.38*	2.24***
	*T. gua* * vs* * T. sor*	2.30***	2.00***	2.78***
	*T. pat* * vs* * T. sor*	4.11***	1.62***	3.45***
*T. gar, T. sor* Arg, *T. sor* BB	*T. sor* Arg * vs* * T. sor* BB	1.21***	1.18***	2.18***
	*T. sor* Arg * vs* * T. gar*	0.61	1.38***	1.27**
	*T. sor* BB * vs* * T. gar*	1.18***	1.88***	1.87***

*P < 0.05; **P < 0.01; ***P < 0.001

**Table 5:** Reclassification of *Triatoma garciabesi* (*T. gar*), *T. guasayana* (*T. gua*), *T. patagonica* (*T. pat*) and *T. sordida* (*T. sor*) performed for shape variables of the head, pronotum and wings. The number and percentage of correctly assigned individuals derived from discriminant function analyses are presented

Species	No. of individuals	*T. gar*	*T. sor*	*T. gua*	*T. pat*	Module
		*n* (%)	*n* (%)	*n* (%)	*n* (%)	
*T. gar*	56	30 (53)	19 (34)	7 (13)	0 (0)	Head
	32	8 (25)	5 (16)	9 (28)	10 (31)	Pronotum
	55	39 (71)	12 (22)	3 (5)	1 (2)	Wing
*T. sor*	82	25 (31)	52 (63)	5 (6)	0 (0)	Head
	73	12 (16)	50 (69)	1 (1)	10 (14)	Pronotum
	91	26 (29)	56 (62)	6 (6)	3 (3)	Wing
*T. gua*	26	1 (4)	3 (12)	17 (65)	5 (19)	Head
	19	2 (11)	4 (21)	12 (63)	1 (5)	Pronotum
	39	1 (3)	2(5)	27 (69)	9 (23)	Wing
*T. pat*	50	0 (0)	0 (0)	7 (14)	43 (86)	Head
	26	7 (27)	1 (4)	2 (8)	16 (61)	Pronotum
	50	2 (4)	0 (0)	3 (6)	45 (90)	Wing

The first two DFA axes performed for the non-allometric component of pronotum shape variation explained 99.74% of total variation (87.65% for axis 1 and 12.07% for axis 2). Mahalanobis distances showed significant differences for all pairs of species except for the comparison between *T. garciabesi* and *T. guasayana* (Table 4, Fig 4b). The NJ tree differed from the tree obtained for head measures. The most similar species were *T. garciabesi* and *T. guasayana* and the most different species was *T. sordida* (Fig. 4b). The factorial map showed that species were partially overlapped in the space of the first two axes (Additional file 1: Figure S1), with *T. garciabesi* being the only species that showed the lowest percentage of correctly assigned individuals (25%, Table 5, Fig. 4b). *Triatoma sordida* was the best classified species according to results for this structure (69%).

Wing shape variation was explained by the first two DFA axes (64% for axis 1 and 31% for axis 2). Mahalanobis distances were significant between all pairs of species (Table 4). The NJ tree was similar to the NJ tree based on head measurements but *T. guasayana* was closer to *T. patagonica* (Fig. 4c). Wing shape exhibited the best percentages of assignment for all species, except for *T. sordida* (Table 5). The factorial map showed that the distribution in the space of the first two DFA axes of *T. garciabesi* overlapped that of the other three species (29% of misclassified individuals), and *T. patagonica* was the species with the best discrimination (90% of correctly classified individuals) (Table 5, Additional file 1: Figure S1).

Comparison of wing CS among the four species showed significant differences (*F*_(3, 240)_ = 9.83, *P* = 0.0001). The *post-hoc* Tukey’s tests revealed significant differences among *T. sordida*, *T. guasayana* and *T. patagonica* and between *T. garciabesi* and *T. patagonica* (all *P* < 0.05)*.*

#### Comparisons among closely related taxa

**Fig. 5:**
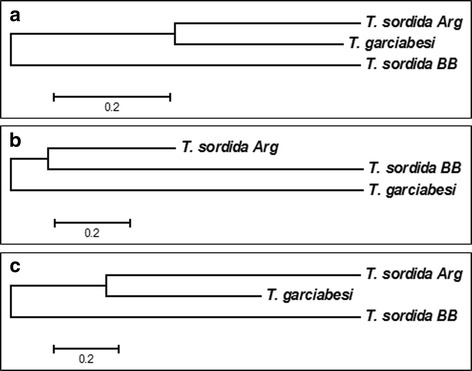
Neighbor-joining trees derived from Mahalanobis distances of shape measurements for the head, pronotum and wings in populations of *T. garciabesi*, *T. sordida* from Argentina and *T. sordida* from Brazil and Bolivia. **a** Head measurements. **b** Pronotum measurements. **c** Wing measurements

The first two DFA axes for head shape contained the whole variation (axis 1: 80.42%; axis 2: 19.58%). No differences were detected in Mahalanobis distances between *T. sordida* Arg and *T. garciabesi* (Table 4). These species formed the pair most closely clustered in the NJ (Fig. 5a). Percentages of correctly assigned individuals were low for *T sordida* Arg, with only 41% of individuals being correctly assigned (Table 6).

**Table 6:** Reclassification of the closely related taxa *T. sordida* from Argentina (*T. sor* Arg), *T. sordida* from Brazil and Bolivia (*T. sor* BB) and *T. garciabesi* (*T. gar*) performed for shape variables of the head, pronotum and wings. The number and percentage of correctly assigned individuals derived from discriminant function analyses are presented

Species	No. of individuals	*T. gar*	*T. sor* Arg	*T. sor* BB	Module
		*n* (%)	*n* (%)	*n* (%)	
*T. gar*	56	32 (57)	15 (27)	9 (16)	Head
	32	21 (66)	9 (28)	2 (6)	Pronotum
	54	36 (67)	13 (24)	5 (9)	Wing
*T. sor* Arg	44	15 (34)	18 (41)	11 (25)	Head
	41	10 (24)	19 (46)	12 (29)	Pronotum
	46	11 (24)	29 (63)	6 (13)	Wing
*T. sor* BB	38	5 (13)	5 (13)	28 (74)	Head
	32	5 (16)	6 (19)	21 (66)	Pronotum
	46	6 (13)	3 (7)	37 (80)	Wing

The first two axes of the DFA performed for the non-allometric component of pronotum shape contained 100% of the variation (axis 1: 76.35%; axis 2: 23.75%). Mahalanobis distances between the three groups were significant, with *T. garciabesi* being the most divergent species in the NJ tree (Fig. 5b). The factorial map showed that *T. sordida* Arg overlapped in the space of the first two DFA axes with the other two groups (46% were correctly assigned) (Additional file 2: Figure S2). *Triatoma sordida* Brazil and Bolivia and *T. garciabesi* both showed 66% of correctly assigned individuals (Table 6).

For wing shape, the first two axes explained 78 and 22% of the total variation, respectively. All pairs of species showed significant differences for Mahalanobis distances (Table 4), and the NJ tree was similar to the one based on head measures (Fig. 5c). The factorial map showed an overlap in the space of the first two axes among the three groups (Additional file 2: Figure S2). The correct assignment of individuals was 67% for *T. garciabesi*, 80% for *T. sordida* Brazil and Bolivia, and 63% for *T. sordida* Arg (Table 6). ANOVA test for wing CS revealed no significant differences among groups (*F*_(2, 144)_ = 0.86, *P* = 0.416).

## Discussion

### Interspecific comparison

Our results showed significant morphological differences among the species of the Sordida subcomplex for the head, pronotum and wing size and shape. We made an important sampling effort in order to cover the morphological variability across almost the entire distribution area of the four species. Moreover, we included the pronotum, a morphological trait that still remains mostly unexplored in studies of morphological variability in the Triatominae.

Wing shape was the only trait that differed in all pairwise comparisons and displayed the lowest classification error in relation to the other two measured structures in all species except for *T. sordida. Triatoma patagonica* exhibited the highest percentage of correctly assigned individuals. This result is in agreement with a previous study where wing shape, rather than head shape, was the character that better discriminated populations of *T. sordida* and *T. garciabesi* [21]. Wing shape has also shown to be less conservative than head shape and presented higher values of Mahalanobis distances within the *Triatoma brasiliensis* species complex [28]. Results of linear and morphogeometric measurements of the head were consistent with those of the wings, with *T. patagonica* having the highest percentage of correctly assigned individuals*. Triatoma sordida* and *T. garciabesi* did not present differences in head shape (Mahalanobis distances were not significantly different) and had a high percentage of incorrectly assigned individuals between them (31 and 34% of individuals were assigned to the other species). Linear and shape pronotum measurements did not show a good assignation percentage for any species. However, pronotum linear measurements improved the percentage of correctly assigned individuals for *T. sordida*, *T. garciabesi* and *T. guasayana* compared to head linear measurements, suggesting pronotum size differences. Pronotum shape was the module that best classified *T. sordida*, but had the highest misclassification error for the other three species. NJ tree topologies showed consistently for wings and head, that *T. sordida* and *T. garciabesi* were more similar to each other than to the other two species of the subcomplex.

Our study showed that *T. patagonica* was the best differentiated species of the subcomplex for head and wing shape, and *T. guasayana* for pronotum linear measurements. In another study, 17 metric variables of different parts of the body were compared among *T. sordida*, *T. guasayana* and *T. patagonica* (*T. garciabesi* was considered a synonym of *T. sordida*). *Triatoma guasayana* and *T. sordida* were completely separated by a discriminant function based on two head measurements, anteocular and second rostral lengths, whereas the three species were discriminated using 14 linear measurements for head, pronotum and abdomen, with a small overlap between *T. guasayana* and *T. patagonica* [14]. Genetic studies based on isoenzymes, chromosome C-heterocromatine banding and mitochondrial sequences have also shown differences between *T. patagonica*, *T. guasayana* and the other species of the Sordida subcomplex [19, 45, 46]. *Triatoma patagonica* is also easily distinguished from the other three species of the subcomplex by its completely dark legs in contrast to the light-colored legs of the other three species [18]. Molecular and cytogenetic analyses and the composition of cuticular hydrocarbons suggest that the taxonomy of the subcomplex should be revised [46–49]. Recent phylogenetic analyses and the chromosomal position of ribosomal genes have placed *T. patagonica* and *T. guasayana* within the Rubrovaria subcomplex and have suggested the reorganization of several subcomplexes within the genus *Triatoma*, including the Sordida subcomplex. In this new proposal, the Sordida subcomplex is composed of six species: *T. sordida*, *T. garciabesi*, *T. jurbergi*, *T. matogrossensis*, *T. vandae* and *T. sordida* from Argentina, the latter being considered a new species [46].

The present results showed that, despite significant morphological differences in wing shape and pronotum linear measures and shape between *T. sordida* and *T. garciabesi*, these species were not well discriminated based on morphological traits. One possibility to explain this result is that individuals from some particular populations were incorrectly identified; another possibility is that morphological characters of some populations differ from the average values for the species due to local adaptation or genetic drift acting in small populations. However, the identity of the misclassified *T. garciabesi* and *T. sordida* individuals by DFA for the different modules was revised, showing that all of them belonged to different populations and the particular identity of each misclassified individual (i.e. individual code number) was not always the same (data not shown). These results suggest that these two species could live in sympatry more often than suspected, even in the same site, with putative hybrids that would hinder morphological identification and morphological delimitation of species [21]. One possibility, suggested to occur in this subfamily (e.g. [1, 50]), is that these two species have speciated very recently and still remain very similar morphologically, with a high degree of phenotypic plasticity across the species distribution area or morphological convergence [50]. Another possibility is that natural hybrids live in sympatry with their parents. This process has been reported to occur in *T. sordida* specimens from La Paz, Bolivia [7].

The taxonomic validity of *T. garciabesi* has been supported by the morphology of the male genitalia, isoenzymatic, molecular, morphometric and cytogenetic traits [17, 19–21, 51]; however, cross-breeding experiments between *T. garciabesi* and *T. sordida* still have not been described and could provide additional evidence for their species status. Also, the search of natural hybrids between *T. sordida* and *T. garciabesi* may shed light on this matter and help understand the possible stage of the process of speciation for these two entities and if there are reproductive isolation mechanisms operating in natural populations.

### Comparison among closely related taxa

The proposed species *T. sordida* Arg differed from *T. sordida* Brazil and Bolivia in all shape traits, but showed the lowest percentage of correctly assigned individuals for shape measurements of the three modules. However, it exhibited the best discrimination for linear measurements of head and pronotum. *Triatoma sordida* from Brazil and Bolivia exhibited, the highest correctly assigned individuals for the three structures, suggesting a small range of intraspecific variation and a better delimitation than *T. sordida* Arg. *Triatoma sordida* from Brazil and Bolivia was the best discriminated for all shape measurements, followed by *T. garciabesi*. In addition, individuals from both *T. sordida* groups were more often confused with *T. garciabesi* than with each other; head shape from *T. sordida* Arg and *T. garciabesi* were not differentiated. NJ trees for wing and head shape illustrated this pattern, with *T. sordida* Arg and *T. garciabesi* being more similar than *T. sordida* from Brazil and Bolivia.

Our results for *T. sordida* Arg and *T. sordida* from Brazil and Bolivia suggest that the proposed cryptic speciation of *T. sordida* Arg is supported not only by chromosome patterns but also by morphological differentiation, since these two entities seem to be delimited by shape morphological traits. Moreover, body size and the pattern of connexivum variation seem to be different (Fig. 2a), although these characters may vary across populations as well as in other species of the Triatominae [52]. In another morphogeometric study of two Brazilian and one Argentine population of *T. sordida* and one population of *T. garciabesi*, the specimens of *T. garciabesi* and Brazilian *T. sordida* were well separated using head and wing shape whereas the Argentine population of *T. sordida* overlapped for wing and head shape with Brazilian *T. sordida* and with *T. garciabesi* [21].

Cryptic speciation in *T. sordida* was first reported for Bolivian populations, where two reproductively isolated cryptic species were living in sympatry in the Bolivian Chaco [20]. Molecular and cytogenetic studies suggest differences in *T. sordida* from Argentina compared with *T. sordida* in the remaining distribution area [7, 19]. Moreover, *T. sordida* cuticular hydrocarbon composition exhibits a heterogeneous pattern, suggesting that it should not be considered a single species [48]. Nevertheless, molecular divergence between *T. sordida* from Argentina and *T. sordida* from Brazil was only 5.3% [7], a similar value to that distinguishing subspecies of *Triatoma sanguisuga* (Le Conte, 1855) [53] and distant populations of *T. patagonica* [52].

For *T. sordida* from Argentina and *T. garciabesi*, the patterns of cuticular hydrocarbons of both species share some similarities [48]. Molecular divergence between *T. sordida* from Argentina and *T. garciabesi* based on mitochondrial DNA was 7%, a similar value to that reported for species of the Brasiliensis subcomplex [7, 30]. Ecological niche modeling revealed clear differences between *T. garciabesi* and *T. sordida* from Argentina and Brazil, with *T. garciabesi* occupying colder and drier areas than *T. sordida* [21]. A low differentiation was achieved between *T. sordida* populations from the wet and dry regions of Argentina (those of the dry region were then described as *T. garciabesi*) [19]. The ecological niche occupied by these two species is not necessarily a criterion to delimit closely related sibling species, since, for example *T. brasiliensis* or *T. dimidiata* complexes occupied different ecological niches across their distribution areas [54, 55].

## Conclusions

To our knowledge, this is the first morphometric study of the species of the Sordida subcomplex that covers almost the entire distribution area of the four species and that includes morphometric traits of the three modules, head, pronotum and wing. Our results suggest that wing shape would be a reasonably good phenotypic marker, since it distinguished the four species traditionally included in the Sordida subcomplex, including the putative new species *T. sordida* from Argentina. *Triatoma sordida* Arg, differed in all measured shape traits from *T. sordida* Brazil and Bolivia; however, additional studies integrating cytogenetic, phenotypic and molecular markers, as well as cross-breeding experiments are needed to confirm the validity of this putative new species.

## Additional files

Additional file 1: Figure S1. Factorial maps in the plane of the two first axes of discriminant function analysis for shape measurements of the head, pronotum and wings in populations of *T. garciabesi*, *T. guasayana*, *T. sordida* and *T. patagonica*. For easy visualization, the lines connect the most external individuals of each population. Centroids were represented with the same symbol that identifies each species. **a** Head. **b** Pronotum. **c** Wings. (TIFF 42083 kb)

Additional file 2: Figure S2. Factorial maps in the plane of the two first axes of the discriminant function analysis for shape measurements of the head, pronotum and wings in populations of *T. garciabesi*, *T. sordida* from Argentina and *T. sordida* from Brazil and Bolivia. For easy visualization, the lines connect the most external individuals of each population. Centroids were represented with the same symbol that identifies each taxa. **a** Head. **b** Pronotum. **c** Wings. (TIFF 43228 kb)
